# Electrospun Materials Based on Polymer and Biopolymer Blends—A Review

**DOI:** 10.3390/polym15071654

**Published:** 2023-03-27

**Authors:** Muhammad Tahir, Silvia Vicini, Alina Sionkowska

**Affiliations:** 1 Department of Biomaterials and Cosmetic Chemistry, Faculty of Chemistry, Nicolaus Copernicus University in Torun, Gagarin 7, 87-100 Torun, Poland; 2 Department of Chemistry and Industrial Chemistry, University of Genova, 16146 Genoa, Italy

**Keywords:** electrospinning, nanofiber, polymer, biopolymer, biomedical, water treatment, supercapacitor

## Abstract

This review covers recent developments and progress in polymer and biopolymer blending and material preparation by electrospinning. Electrospinning is a technique that is used to produce nanofibers to improve the quality of membranes. Electrospun nanofibers are highly applicable in biomedical sciences, supercapacitors, and in water treatment following metal ion adsorption. The key affecting factors of electrospinning have been checked in the literature to obtain optimal conditions of the electrospinning process. Future research directions and outlooks have been suggested to think about innovative ideas for research in this field.

## 1. Introduction

**Figure 1:**
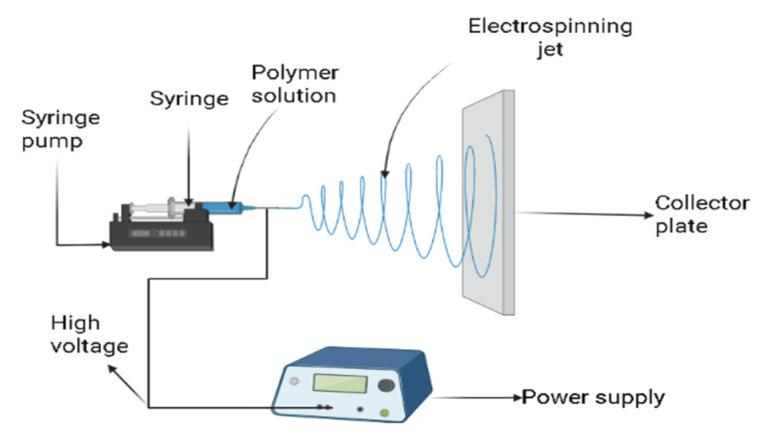
Electrospinning setup.

The production of polymer fiber with an electric field has been known since 1930 [1]. This process of polymer fiber production was named electrostatic spinning or electrospinning in the 1990s [2]. Electrospinning is the facile approach used for the generation of micro/nanofiber mats, and the diameter of the generated fibers can be adjusted in the range of nanometers to microns [3]. Electrospinning has an advantage because of its simple design [4]. It consists of three main components, which are the feeding unit, the collector plate, and the high-voltage power supply. Polymers in melted or completely dissolved form are introduced into the feeding unit. The polymer solution is most commonly used rather than melted polymer solution because it does not require heating. The high voltage is applied to overcome the surface tension in the charged polymer [5,6]. When an electrical potential is increased, the Taylor cone is formed at the top of the capillary, and it causes the formation of the charged jet from the apex of the Taylor cone [7]. The scheme of electrospinning setup is presented in Figure 1.

As mentioned above, there are two major types of electrospinning, which are melt electrospinning and solution electrospinning [8]. Melt electrospinning helps to obtain a higher output with safety, and melt electrospinning requires less energy consumption. Melt electrospinning produces fibers with excellent mechanical, electrical, and optical properties [9]. Melt electrospinning is cost-effective because it does not use solvents for application and recovery. Melt electrospinning produces nanofibers with a diameter in a range of 10 to 50 μm [8]. Solution electrospinning is relatively easy due to its simple design because no additional assembly is required. Solution electrospinning produces fibers in a range of 100 to 1000 nm. Thermally unstable polymers are the best choice for solution electrospinning [9]. Solution electrospinning is responsible for most of the nanofiber’s production, and it is utilized for laboratory research and industrial production of the nanofibers [10]. 

Various types of materials are used in electrospinning to produce electrospun nanofibers. The well known materials used are organic polymers, small molecules, colloidal particles, and composites. Organic polymers can be applied either in melted form or in solution form. Organic polymers are usually dissolved in solution to be used in solution form [11]. The solubility parameter plays an important role in the formation of a homogenous solution [12,13]. Small molecules are mainly derivatives of amphiphiles and cyclodextrin [14,15]. Lecithin was the first amphiphile used in electrospinning, and lecithin is a combination of phospholipids and neutral lipids [16,17,18]. Colloidal particles in electrospinning are produced by hydrolysis and condensation of metal alkoxides or metal salts [13]. The silica solution was prepared by using tetraethyl orthosilicate (TEOS), distilled water, ethanol, and HCl at a temperature of 80 °C for 30 min, and this silica solution was used in electrospinning [19]. Composites prepared due to the addition of sol–gel were studied extensively for solution electrospinning. The sol−gel reactions were carried out in a jet with access to surrounding air [20]. There are many reports in the literature regarding electrospinning.

This review mainly focused on electrospinning, its techniques and affecting factors, and its recent progress and applications. Electrospinning techniques, such as needle-based electrospinning and needleless electrospinning, are being used for fiber production. Needle-based electrospinning is used for small-scale production of fibers. Needleless electrospinning is used for industrial applications. Electrospinning is a versatile and interdisciplinary technique, so it has applications in various fields, such as biomedical sciences, water treatment, and supercapacitor research. 

## 2. Electrospinning Techniques

### 2.1. Needle-Based Electrospinning

There are two standard types of needle-based electrospinning setup, either vertical or horizontal. This classification is opted due to geometric-arrangements of collecting plate and ejecting capillary [21].

#### 2.1.1. Single-Nozzle Electrospinning

The spinneret used in single nozzle electrospinning is a single needle, and it is simplest form of needle-based electrospinning. In single-nozzle electrospinning, the formation of the Taylor cone takes place on the top of the capillary [22]. A single polymer or polymer blend can be used for the production of uniform nanofibers [23]. Side-by-side electrospinning is a modified form of single-nozzle electrospinning [24]. A nanofiber produced by side-by-side configuration is termed as Janus fiber [25].

#### 2.1.2. Multi-Nozzle Electrospinning

Multi-nozzle electrospinning is used to enhance nanofiber yield and the production rate. The nanofiber production rate is enhanced by introducing the number of needles, holes, and tips. The operating procedure of a multi-nozzle is similar to a basic electrospinning system, and it involves the interaction of Coulomb forces and electric field forces [26].

#### 2.1.3. Coaxial Electrospinning

Coaxial electrospinning leads to the production of core-sheath fibers. In coaxial electrospinning coaxial spinneret is used, this coaxial spinneret usually contains an inner or outer needle [27]. Solution pairs are used in coaxial electrospinning for the production of fibers, such as hollow fiber or core-sheath fibers [28]. The fiber produced by coaxial electrospinning shows a variety of composite properties [29,30].

#### 2.1.4. Triaxial Electrospinning

Three polymer solutions are introduced by spinneret to compound the Taylor cone, and this technique of electrospinning is termed triaxial electrospinning. The loading of polymer with nanoparticles is common in triaxial electrospinning. In a dual drug delivery system, the triaxial fiber is considered to supply two different drugs due to its isolation layer [31]. The variation in mechanical strength and hydrophobicity of fibers can be dealt through triaxial electrospinning [32].

#### 2.1.5. Electroblowing

The modification of electrostatic spinning by an introduction of flowing air blown to boost the fiber production process is known as electroblowing, and this flowing air blown is introduced to the spinning nozzle. Variation in flowing air properties leads to variation in the properties of fibers being produced, and properties of flowing air, such as air velocity adjustment, help to obtain fibers of desired requirements [33].

#### 2.1.6. Centrifugal Electrospinning

Centrifugal electrospinning utilizes centrifugal force and electrostatic forces for fiber production. Centrifugal electrospinning devices are mainly composed of components such as the servo motor, feeding plate, holder, high voltage, collecting plate, and rotating nozzle [34]. Rihova et al. [35] reported that fiber diameter is decreased by an increase in relative humidity level during centrifugal electrospinning. Centrifugal electrospinning is used to achieve a higher efficiency of fibers [36].

### 2.2. Needleless Electrospinning

Nanofiber production on a larger scale is achieved by needleless electrospinning. The quality of fiber by needleless electrospinning is better than needle-based electrospinning [37]. On other hand, fiber production by needle-based electrospinning is achieved by increasing the number of nozzles. 

#### 2.2.1. Bubble Electrospinning

Basic electrospinning is dependent on the spinnability of polymer solutions, and bubble electrospinning depends on geometrical properties, such as the size of formed bubbles. Rui et al. [38] carried out an experiment of polyvinyl alcohol and water for fiber production and produced environment friendly fiber with a diameter of 46.8 nm.

#### 2.2.2. Splashing Electrospinning

Splashing electrospinning has modified the polymer solution supply system, and this polymer solution is applied in the splashed form to the metal roller spinneret. In splashing electrospinning, the solution distributor is arranged on top of a metal roller, and the splashing of the polymer solution from the solution distributor leads to a gravitational field due to height difference [39].

#### 2.2.3. Edge Electrospinning

Edge electrospinning is used for batch operation, and in edge electrospinning, a bowl fill is used for polymer solution [40]. Edge electrospinning is highly applicable to those polymer solutions having a difference in viscosities [41].

#### 2.2.4. Two-Layer Fluid Electrospinning

In two-layer fluid electrospinning, the upper layer is referred to polymer solution, while the lower layer is applied to ferromagnetic suspension. A coil or wire is used to generate a magnetic field, and two layers are charged with a magnetic field. The addition of an electric field leads to the formation of nanofiber in an upward direction by a motion of the lower layer disturbing the upper layer of polymer solution [42].

## 3. Affecting Factors of Electrospinning

The important factors responsible for the electrospinning process can be classified into two categories, which are either polymer solution process conditions or process equipment conditions. Environmental factors also have an impact on the electrospinning process [8]. 

### 3.1. Polymer Solution Affecting Factors

**Figure 2:**
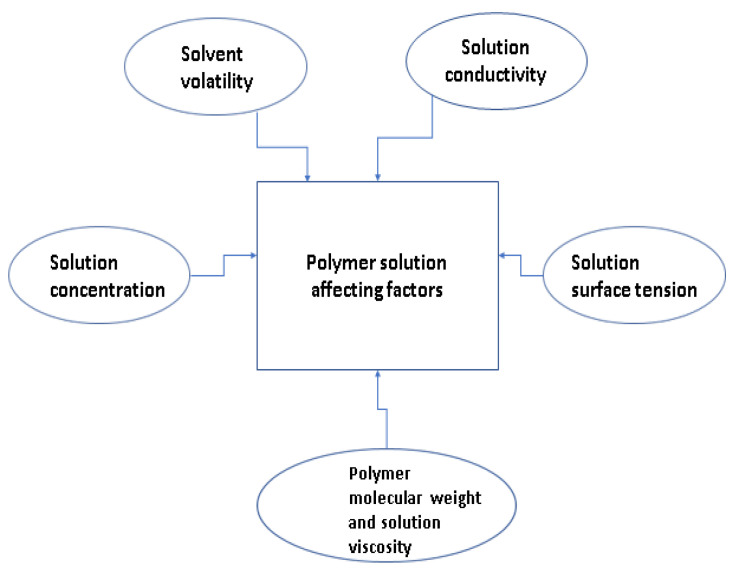
Polymer solution affecting factors.

Important affecting factors regarding polymer solution are polymer molecular weight and solution viscosity, solution concentration, solvent volatility, solution conductivity, and solution surface tension [43]. Polymer solutions affecting factors have been shown in Figure 2. 

#### 3.1.1. Polymer Molecular Weight and Solution Viscosity

The molecular weight of the polymer is a representation of presence of polymeric chain entanglements, and polymers with lower molecular weight also contain polymeric chain entanglements to ensure the solution viscosity requirements. Higher molecular weight is not always the requirement for electrospinning if the polymer has enough interchain connectivity [44]. Molecular weight can affect these parameters, such as viscosity, surface charge density, and surface tension [45]. Lin et al. [46] measured the viscosity of colloidal solutions with a viscometer at room temperature and 65% humidity. The colloidal solution of polymethylmethacrylate with higher viscosity resulted in the unstable jet state, and it caused the fabrication of fibers with banded structure. Zadeh et al. [47] stated that the viscosity of polyvinyl alcohol (PVA) with solutions of less than 5% was very low and independent of shear rate, and it follows Newtonian flow behavior. When PVA solution of 6–10% was prepared, it resulted in a higher viscosity, and it followed shear thinning behavior. The molecular weight of PVA in the above-mentioned research was 7200.

If the solution concentration is kept constant during electrospinning, then an increase in molecular weight will result in the formation of smooth fibers, and a decrease in molecular weight will lead to bead formation [48]. 

#### 3.1.2. Solution Concentration

There is a direct effect of polymer concentration on solvent evaporation and solution viscosity. If a polymer is in low concentration, then electro-spraying must be performed instead of electrospinning [49]. If a polymer solution with a higher concentration is used, then fibers of larger diameter are produced as compared to a diluted solution [50]. Zeitoun et al. [51] investigated two different concentrations of polyvinylidene fluoride (PVDF)—14wt% and 16wt%—and observed that 16wt% solution of PVDF generated few beads with membrane structure. The reason for the generation of beads with membrane structure is that polymer induces long-chain entanglements, which are responsible for the continuity of the jet during the electrospinning [52].

#### 3.1.3. Solvent Volatility

Solvent volatility plays an important role in influencing the electrospinning process. If a solvent is highly volatile, then the collection of dry fibers can be facilitated. When solvent with higher volatility starts evaporating, it may cause needle clogging, which results in the polymer solidification at the needle tip. If the solvent has lower volatility, then the production of fine fibers is possible because the solvent does not clog at the needle tip [53,54].

#### 3.1.4. Solution Conductivity

Solution conductivity plays a pivotal role in controlling the diameter of nanofibers. The charge density of polymer solution can be determined by solution conductivity, and charge density is responsible for controlling bending and repulsion extent during the electrospinning process. Uyar et al. [55] conducted experiments with dimethylformamide (DMF) as a solvent for polystyrene polymer solutions with different solution conductivities. The results showed an inverse relationship between solution conductivity and fiber diameter, and higher solution conductivity resulted in smaller fiber diameter. 

#### 3.1.5. Solution Surface Tension 

Wang et al. [56] measured surface tension by using a tensio-meter. The polymer solutions that have the highest surface tension confirm the fabrication of fibers with beads, and polymer solutions with the lowest surface tension result in the inhabiting formation of beads and tend to increase fiber diameter [57]. 

### 3.2. Equipment Parameters Affecting Factors

**Figure 3:**
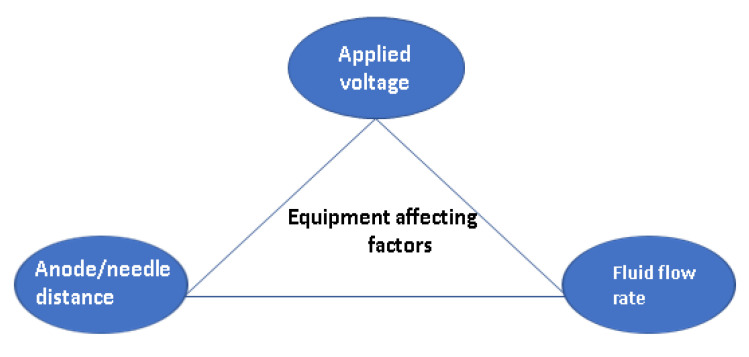
Equipment affecting factors.

The affecting parameters regarding electrospinning equipment are applied voltage, anode/needle distance, and fluid flow rate [58]. These parameters are summarized in Figure 3 and characterized below. 

#### 3.2.1. Applied Voltage

The formation of a stable cone from solution is dependent on a specific range of applied voltage. The pendant can be formed at the exit of the nozzle due to the dripping of two solutions in the case of low voltage. If voltage is increased to a maximum value, it also leads to clogging due to fluid jets inside the capillary [27,59]. The increase in voltage causes a decrease in nanofiber diameter [60]. 

#### 3.2.2. Anode/Needle Distance

There is a direct effect of a needle-to-collector distance on the solution electric field. When needle-to-collector distance is increased, then an exponential decrease in volume charge density is observed. It is noted that, with a 5 cm to 20 cm needle-to-collector distance, the nanofiber size range was from 210 nm to 310 nm. The increase in nanofiber size was not remarkable with an increase in anode-to-collector plate distance [61].

#### 3.2.3. Fluid Flow Rate

The flow rate of fluid has an impact on the shape/size of the droplet formed at the tip of the spinneret, nanofiber diameter, morphology, path of the jet, and transformation of the charged droplet into Taylor cone [62,63]. Zong et al. [64] stated that, to establish a stable Taylor cone minimum value of a solution, flow rate is required to form a drop of the polymer at the tip of the needle. Results showed that the flow rate could vary the fiber diameter. When the volumetric flow rate is increased, then an increase in a solution being delivered to the tip of the needle is also observed, which results in the formation of larger diameter nanofibers [65,66].

### 3.3. Environmental Affecting Factors

**Figure 4:**
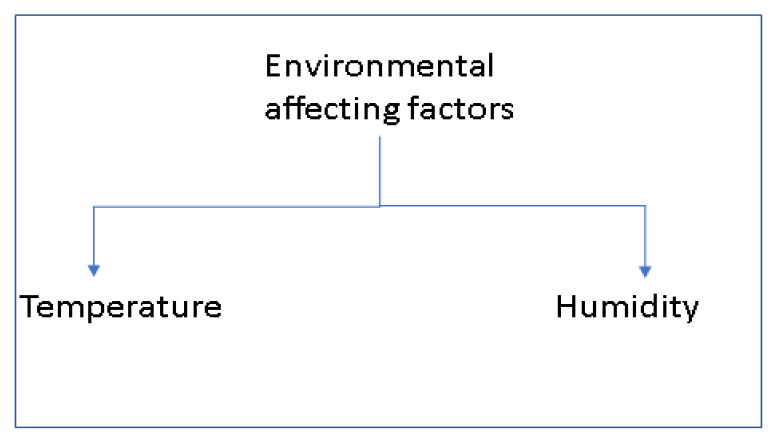
Environmental affecting factors.

Nanofiber formation can be affected due to environmental factors during an experiment. Temperature and humidity are key environmental factors that influence electrospinning conditions (Figure 4) [67].

#### 3.3.1. Temperature 

Vrieze et al. [68] carried out an experiment to observe the effect of temperature on the solution of cellulose acetate (CA) and polyvinyl pyrrolidone (PVP). They noticed that temperature had an affecting on two parameters, and these two parameters can play a role in affecting the average fiber diameter. These two parameters are solvent evaporation rate and viscosity of the solution. They mentioned that temperature has a direct impact on solvent evaporation rate, and temperature has an inverse impact on solution viscosity. 

#### 3.3.2. Humidity

During electrospinning, alteration in humidity can affect fiber morphology. Casper et al. [69] observed increasing humidity levels during electrospinning results in smaller circular pores on the fiber surface. Megelski et al. [70] reported that polymers dissolved in volatile solvents at a high level of humidity resulted in fiber production with condensed water on the surface of the fiber. Li and Xia [71] stated that higher humidity facilitates fiber discharge. Low humidity enhances solvent evaporation rate, and high levels of humidity cause issues with solvent evaporation. Nanofiber morphology is obtained with an adjusted level of humidity [72]. Low humidity caused an increase in charge density, and it leads to air breakdown [73].

## 4. Electrospun Nanofibers in the Biomedical Field

**Figure 5:**
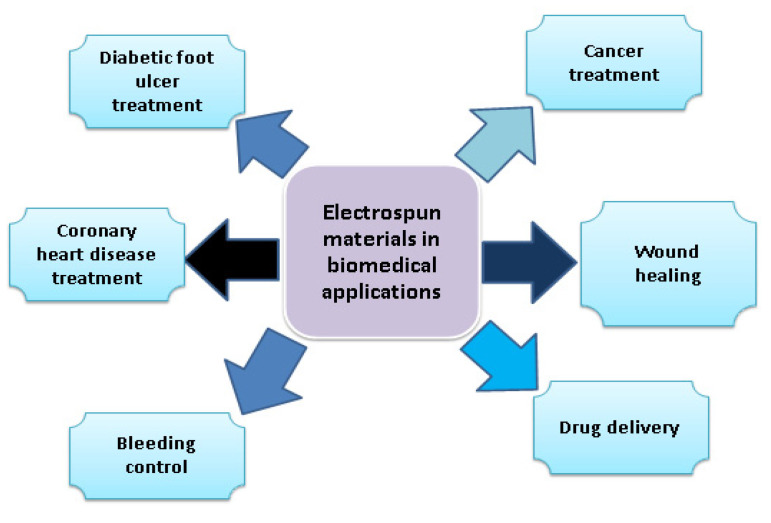
Biomedical applications of electrospun materials.

Progress in the biomedical field is based on the development of biomedical applications, such as the evaluation of bio-compatibility of implants, regenerative tissue engineering, drug delivery systems, and imaging devices (Figure 5). Biomaterials play a key role in the development of biomedical devices, such as bone implants, contact lenses, stents, artificial hearts, tissue adhesives, surgical sutures, and dialysis membranes [74,75,76]. Materials for biomedical applications need to be bio-compatible, and they need to be not harmful to living tissues [77]. Bio-compatibility tests, such as in vitro and in vivo tests, are compulsory for the development of medical devices [78], for example, for coronary heart disease.

Coronary heart disease (CHD) is linked with blockage of coronary arteries, and it accounts for almost 50% of cardiovascular deaths. Blood is supplied to the heart by these arteries [79]. CHD can be treated with help of tissue-engineered vascular grafted materials [80]. Synthetic polymer application in tissue-engineered vascular grafts has attracted great attention to repair injured blood vessels due to their strong structural support [81]. Polyurethane (PU) has elastic mechanical properties, bio-degradable properties, and excellent biocompatibility properties [82].

For biomaterials preparation, chitosan is widely used. Chitosan is a biopolymer derivative of chitin, and it is widely used in tissue engineering and drug delivery systems. Chitosan nanoparticles were synthesized and loaded with PU to form electrospun vascular membranes [83,84]. For elastin and chitosan blended and loaded with PU, the results showed that blending of chitosan and elastin improved the hemocompatibility of PU [85]. The binding of red blood cells with chitosan led to rapid blood coagulation. Chitosan electrospun nanofibers are used to control bleeding, and they are also used in wound healing and for dryness of wounds. The chronic complication of diabetes is diabetes foot ulcer, which causes long-term hospitalization. Chitosan-based nanofibers are preferred to be used for diabetic foot ulcers due to chitosan properties, such as ease of use and bio-degradability [86,87].

There is also a report on other blends for electrospinning. Sen et al. [88] prepared a blend of two natural polymers (hibiscus leaves mucilage and pectin) and a synthetic polymer (polyvinyl alcohol) by electrospinning technique for the wound healing process. It was found that hibiscus leaves, mucilage, and pectin in nanofiber scaffolds have been effective for wound treatment. The addition of synthetic polymer polyvinyl alcohol enhanced the mechanical properties of the scaffold, and it increased the thermal stability of the fiber, and it also added polarity to the film. Mucilage-pectin-PVA (volume ratio) 1:1:8 led to fiber formation with no droplets. 

Graphene oxide (GO) and egg white proteins (EWPs) have applications in wound healing. Wang et al. produced wound dressing of composite GO/EWPs/PVA, and they prepared this blend with varying amounts of GO. The varying amounts were 0, 0.5, 1, and 2 mg/mL, so they used the electrospinning and crosslinking technique. They observed that the addition of graphene oxide boosted wound healing activity. Composite fibrous wound dressing with 1 mg/mL has shown excellent physiochemical properties and wound healing effects, and it also has the ability for tissue regeneration [89].

Sodium alginate (SA) and polyvinyl alcohol (PVA) polymers are famous for biomedical applications, and a blend of these two polymers can enhance the biological, mechanical, and physical properties of the wound healing material. This polymer blend can be cross-linked with Ca^+2^ to enhance wound healing activity. This cross-linking also adds antioxidant and anti-microbial properties to the polymer blend of SA and PVA [90].

Gonçalves et al. [91] analyzed chitosan/polyvinyl alcohol/glycerol electrospun nanofibers to be applied in skin care and found the best sample with chitosan: polyvinyl alcohol 1:3.5 ratio. The conclusion has been drawn with the suggestion that the quality of nanofibers is based on the degree of hydrolysis of polyvinyl alcohol (PVA), rather than its molecular weight. The addition of glycerol to chitosan/PVA blend plays a role to lessen the solubility of this blend, and it also weakens the interaction of molecules [92].

Tajul et al. [93] carried out the fabrication of nanofibrous scaffold of keratin (5% *w*/*v*) and chitosan (2% *w*/*v*) with polyvinyl alcohol (10% *w*/*v*). Keratin/PVA and chitosan/PVA nanofibrous scaffolds have been studied for biomedical applications, so it was a unique idea to fabricate PVA/keratin/chitosan. These three-dimensional nanofibrous scaffolds (3DENs) have the potential to be used for biomedical applications according to the results.

Electrospinning and film casting methods are used for poly-_L_-lactic acid (PLA) and chitosan blends [88,94]. PLA/chitosan blend acts as wound dressing material, and it can form an effective barrier against bacteria [95]. It has excellent mechanical properties, and these properties help in bone fracture healing in orthopedic and oral surgery [96,97]. This blend also has applications in the field of tissue engineering [98], and it can be utilized in nerve tissues [99] and for drug delivery [100,101].

There are two major ways of drug delivery by electrospinning, either blending of drug with a polymer solution or chemically grafting the drug on the molecular structure of the polymer, and chemical grafting is possible with chemical bonding [102]. Rychter et al. [103] produced fibers of poly-ε-caprolactone (PCL) and a composite of poly-ε-caprolactone (PCL) and cilostazol (CIL) by electrospinning, and they noticed that cilostazol (CIL)-loaded nano-membranes have vascular implant applications. Luo et al. [104] developed an implantable micelle depot by blending camptothecin (CPT)-containing promicelle polymers (PM _CPT_), polyethylene oxide (PEO), and polyethylene glycols (PEG)-poly lactide (PELA). Implantable micelle depot had great potential for cancer therapy.

Cancer is a threat to human health [105], and a major issue associated with cancer therapy is the off-target effect of chemotherapy [106]. Xie et al. [107] used blend electrospinning to fabricate fibers of polyvinylidene fluoride (PVDF) and incorporated it with disulfiram (DSF). Characterization techniques, such as EDS, FTIR, and SEM, have been employed to verify the incorporation of DSF. PVDF-SDF drug release behavior was checked using high-performance liquid chromatography (HPLC). They found that the PVDF-SDF scaffold has great potential to be used in cancer treatment.

Poly-ε-caprolactone (PCL) is a semi-crystalline polymer, and it is known for its biomedical applications. The melting point of PCL is 60 °C, and it is quite low. The tensile strength of PCL is higher, and it is biodegradable [108,109]. Techniques used to prepare PCL/chitosan blends are electrospinning [110], cast solution method [111], semi-interpenetrating polymer networks [112], and hydrogel formation [113].

Poly(N-vinyl-2-pyrrolidone) (PVP) is hydrophilic, and it is non-toxic, so it is considered an important component for the development of biomaterials. Processing of PVP is difficult due to its rigidity and fragility [114]. The addition of chitosan to PVP causes modification in the physio-chemical properties of PVP, especially in bio-compatibility [115]. Chitosan/PVP blend forms a homogeneous phase, and it is a result of hydrogen bonding. The main reason for the enhancement of chitosan/PVP blend properties is the result of their high-level miscibility [116]. Grant et al. [117] carried out experiments by electrospinning composed of 4% chitosan and 6%, and they concluded that they did not notice a change in the properties of chitosan and PVP. So, it resulted in fine fibers being used in drug delivery.

Poly-γ-glutamic acid (PGA) has applications in tissue engineering due to its bio-compatibility [118]. PGA/chitosan blend is prepared to overcome PGA intermediate degradation products when it is hydrolyzed in acidic media [119]. PGA has a huge amount of carboxyl groups, as it is considered a polyanionic biopolymer [120]. The addition of chitosan with PGA causes the production of biomaterial, which has excellent hydrophilicity and cyto compatibility properties [121]. Kim et al. [122] carried out electrospinning of poly γ-glutamic acid (PGA) and chitosan oligomer to prepare polyelectrolyte complex against moisture in the air.

Munj et al. [123] developed a ternary blend of poly methyl methacrylate (PMMA)-poly caprolactone (PCL)-gelatin by electrospinning for biomedical applications. PMMA added strength to the scaffold and enhanced degradation time. Adhesion of proteins and cells is achieved by the addition of gelatin, and it makes the blend surface bioactive. Structural properties of the blend are maintained because of PCL.

Zahra et al. [124] analyzed a polymer blend of cellulose acetate (CA) and ethyl cellulose (EC), and they loaded this polymer blend with ketoprofen (KET) by electrospinning. EC has hydrophobic properties, and it is non-toxic. EC has a stability of the storage, and it has inert nature [125]. CA can be used as electrospun nanofiber mats. It also has applications in separation processes [126], in transdermal drug delivery systems [127], and in the manufacturing of edible nanofibrous thin films [128]. KET is a non-steroidal anti-inflammatory drug, so it is used to relieve pain connected with tissue injury [129].

## 5. Electrospun Nanofibers in Supercapacitor

Society and industrial energy requirements are dependent on a huge amount of consumption of depleting fossil fuel sources, such as coal and oil and gas [130,131]. Researchers nowadays focus on the development of portable electrochemical energy storage devices with maximum energy power [132]. Consumption of fossil fuels is the reason for carbon accumulation in the natural cycle [133,134,135]. There are various portable energy storage devices, such as batteries, traditional capacitors, and supercapacitors. Supercapacitors are superior due to their advantages, such as long cyclic stability, fast charging speed, and maximum energy power [136,137,138,139]. The major issue with supercapacitors is low energy density [140]. Electrospinning can be used also in the field of supercapacitors.

Wang et al. [141] activated porous carbon nanofibers (PCNFs) using a blend of high-amylose starch (HAS) and polyacrylonitrile (PAN) by electrospinning. Carbon materials are considered highly applicable as electrode materials for supercapacitors [142,143]. They observed that carbon materials achieved higher micro-porous pores at a specific area of 1204 m^2^g^−1^. They suggested HAS for in situ activation of PCNFs for supercapacitors to achieve a high level of performance [141].

Polyimide (PI) and polyvinyl chloride (PVC) blend are prepared by electrospinning, and electrospun fibers of PI/PCL show a specific level of phase separation. The reason behind this level of phase separation is an incompatibility of PI and PVC in N and N′-dimethylformamide (DMF). Carbon nanofibers of PI/PVC having mesoporous structure served as an electrode, and it was proven to possess fast ion mobility/diffusivity [144].

Abeykoon et al. [145] derived electrode material for supercapacitors from a polymer blend of polyacrylonitrile (PAN) and polymethyl methacrylate (PMMA). PAN acted as carbonizing polymer, while PMMA was utilized as a sacrificial polymer. Electrospun nanofiber mats were prepared by dissolving polymer blend into dimethyl formamide (DMF). Electrospun nanofibers were further carbonized, and their activation was completed with CO_2_ at 1000 °C, which converted PAN into carbon and PMMA decomposed with generating pores.

Wang et al. [146] used this approach to blend polysulfone (PSF) and polyacrylonitrile (PAN) by electrospinning, and in the next step, the polymer blend is carbonized. They observed that using PSF resulted in increased mesopore contents, inter-fiber connection, degree of graphitization, conductivity, and specific area of carbon nanofibers. The optimal result was obtained with 20% PSF, and, when the PAN/PSF electrode material is used, then the super capacitor achieved specific high capacitance as an electrode material, and the prepared supercapacitor showed a specific capacitance as high as 289 F/g at the scan rate of 10 mV/s and 257 F/g at the current density of 0.25 A/g. The device achieved excellent cycling stability (100% capacitance retention after 6000 cycles) and large energy capability.

Yu et al. [147] demonstrated single-needle electrospinning to prepare novel dendrite-like 3D MgCo_2_O_4_/C for supercapacitor applications. They noticed that carbonization temperature affected the structure and morphology of the product. The dendrite-like three-dimensional MgCo_2_O_4_/C was prepared with a larger surface area of 314.027 g/m^2^, and it achieved unique structural properties and excellent chemical properties.

Manganese oxide is considered a promising pseudo-capacitive material due to its cost-effectiveness, variation of oxidation states, and high theoretical capacitance. Electrospinning is applied to incorporate MnO nanoparticles with carbon nanofibers (MnO-CNFs). Carbonization is controlled during electrospinning, and it is applied as an electrode in a supercapacitor. A supercapacitor with MnO-CNF electrode obtained a high specific capacitance of 246 F g^−1^ at 0.5 A g^−1^ in a three-electrode system. Supercapacitor device with MnO-CNF electrodes achieved a maximum power density of 5000 W kg^−1^, maximum energy power of 14 W h kg^−1^, and excellent cycling stability (97.5% retention after 10,000 cycles) [148]. 

Flexible SrCexTi_1−x_O_3_ nanofibers films (SCTO-x) was prepared with a perovskite structure by using electrospinning combined with low temperature (600 °C) sintering technology. By Ce doping, the electrochemical properties of electrodes to store energy were improved. Investigation of SCTO-x samples results showed that optimal electrochemical performance is achieved by sample SCTO-0.3 in 1 M in Na_2_SO_4_ solution. The device achieved an outstanding capacitance of 174.2 F · g^−1^ at 1.25 A · g^−1^ [149].

## 6. Electrospun Nanofibers in Metal Ion Adsorption

**Figure 6:**
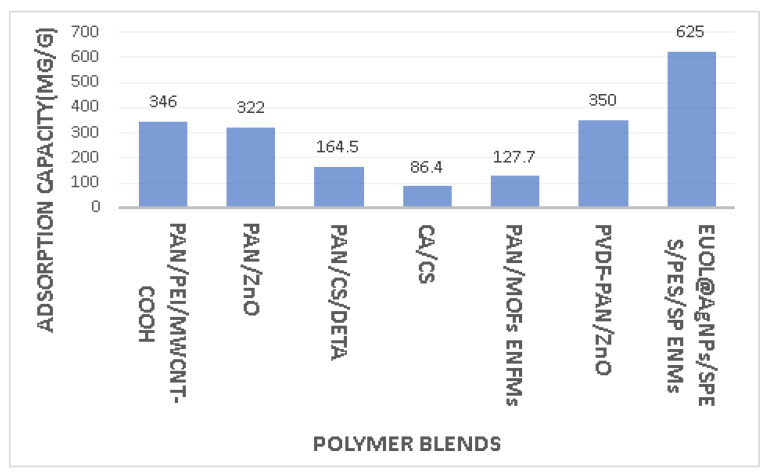
Heavy metal adsorption capacity for polymer blends.

Water is important for the continuation of life, and water is required for various sectors, such as industry, agriculture, and hydropower generation [150]. Toxic contaminants discharged into water bodies are the main reason for water pollution [151]. Industrial waste from electroplate, leather, textile, and printing industries, containing organic dyes and heavy metals discharged into water, is a potential hazard to the environment [152,153,154]. Organic and inorganic contaminants from wastewater can be eliminated by fabricated electrospun nanofibers due to adjustable wettability, surface morphology, and higher length-to-diameter ratio [155]. Predoi et al. [156] used hydroxyapatite to remove lead ions from an aqueous solution. There are also reports about polymers used for elimination of contaminants from water (Figure 6).

Guo et al. [157] prepared composite nanofiber membranes (CNMS) by electrospinning of polymer blend composed of polyacrylonitrile (PAN)/polyethyleneimine (PEI)/carboxylated multi-walled carbon nanotube (MWCNT-COOH). CNMs achieved separation efficiency of more than 83% and represented high flux (800 L m^2^ h^−1^) in water–oil emulsions. CNMs also have excellent adsorption capacity for heavy metal ions. The adsorption capacity for Pb^+2^ by CNMs was reported to be 346 mg/g.

Haddad et al. [158] checked the incorporation effect of electrospun polyacrylonitrile (PAN) nanofibers with different amounts of ZnO nanoparticles on the adsorption of lead (PbII) and cadmium (CdII) from aqueous solution. The best adsorption capacities experienced at the surface of PAN/ZnO composite nanofibers for lead (PbII) ions and cadmium (CdII) ions were 322 and 166 mg/g. Results indicated that the incorporation of PAN/ZnO enhanced the adsorption capacity of lead (PbII) ions by 261% and cadmium (CdII) ions by 167%. 

Alharbi et al. [159] produced a bilayer nanofibers membrane of polyacrylonitrile (PAN) with ZrO_2_ or TiO_2_ metal oxide with a layer of chitosan by electrospinning. They investigated the adsorption capacity of this bilayer nanofiber membrane against lead (PbII) and cadmium (CdII) ions. This bilayer nanofiber membrane increased the adsorption capacity of cadmium by 405% more and of lead by 102% more than a single layer of PAN/MO. 

Zhang et al. [160] incorporated electrospun polyacrylonitrile (PAN)/chitosan (CS) with diethylenetriamine (DETA) to obtain a nanofiber membrane. The adsorption capacity of the nanofiber membrane reached up to 164.5 mg/g for copper (Cu^+2^) ions, with specified conditions of 46% weight gain rate and pH 5-6. This nanofiber membrane can represent concentrations of copper (Cu^+2^) ions with different gradient colors. 

Xu et el. [161] mentioned that cellulose and chitosan are biocompatible, so they can also be used in biomedical applications. Li et al. [162] fabricated cellulose acetate (CA)/chitosan (CS) ultrafine nanofibrous membrane by electrospinning to efficiently remove aquatic copper ions. The diameters measured for conventional and ultrafine nanofibers with 50% and 30% chitosan were 56.22 nm and 37.28 nm respectively. The 30% CS-loaded CA/CS biocompatible nanofiber membranes at a pH level of 5 showed an adsorption capacity of copper ions 86.4 mg/g.

Talukder et al. [163] prepared eucommia ulmoides leaf extract stabilized silver nanoparticles (EUOL@AgNPs) incorporation with sulfonated polyether sulfone (SPES)/polyethersulfone (PES) electrospun nanofiber membranes (SP ENMs) by electrospinning. They conducted experiments to study the removal of lead (PbII) and cadmium (CdII) from aqueous solutions. They observed maximum adsorption capacity for Cd(II) and Pb(II) was 625 and 370.37 mg/g respectively at neutral pH.

Polyelectrolyte fiber was produced by electrospinning of polyacrylic acid (PAA) and polyallylamine hydrochloride (PAH) complex solutions, which was then followed by thermal crosslinking. When the pH level was 3.4, fiber mats showed 63% removal of the tested heavy metals (Pb, Cd, and Cu), and 98% removal was observed at pH 7.4 from synthetic metal solutions, and this confirmed that the removal of Pb, Cd, and Cu by electrospun fiber is effective at higher pH [164].

Wu et al. [165] prepared a nanofibrous membrane by electrospinning chitosan (CS)/ polyvinylpyrrolidone (PVP)/Polyvinyl alcohol (PVA) to remove organic matter and heavy metal ions from water. CS/PVP/PVA nanofibrous membrane represented a porous and uniform nanofibrous structure, which has an average diameter of 160 nm and water permeability of 4518.91 L⋅m^−2^⋅h^−1^⋅bar^−1^. The evaluation was made for CS/PVP/PVA nanofibrous membrane against these ions, Cu(II), Ni(II), Cd(II), and Pb(II), and separation was also checked for methylene blue (MB) and malachite green (MG).

Metal–organic frameworks (MOFs) are a kind of porous adsorbent, and they are well known for the removal of Cr(IV) from water. Iron-based MOFs were synthesized by solvothermal technique, and they were co-electrospun with polyacrylonitrile (PAN) to obtain composite electrospun nanofibrous membranes (PAN/MOFs ENFMs). PAN/MOFs ENFMs experienced adsorption of Cr(IV) from water, and adsorption capacity was noted at 127.70 mg/g at a pH level of 4. The fraction of Cr(IV) ion was converted into Cr(III), which was less toxic as compared to Cr(IV) [166].

Assaifan et al. [167] used coaxial electrospinning to encapsulate polyvinylidene fluoride (PVDF) with polyacrylonitrile (PAN) loaded with zinc oxide (ZnO). Various electrospun nanofibrous membranes were used to adsorb heavy ion metal, but the problem was regarding its mechanical strength. The main concept to encapsulate PVDF with PAN is to mechanically strengthen the nanofibrous membrane, and the loading of ZnO is used to enhance the adsorption capacity of cadmium ions. The results represented the adsorption capacity of PVDF-PAN/ZnO up to 350 mg/g, and PVDF-PAN showed an adsorption capacity of up to 200 mg/g for cadmium ions [167].

## 7. Research Comparison of Electrospun Material in the Field of Polymer Blends

**Figure 7:**
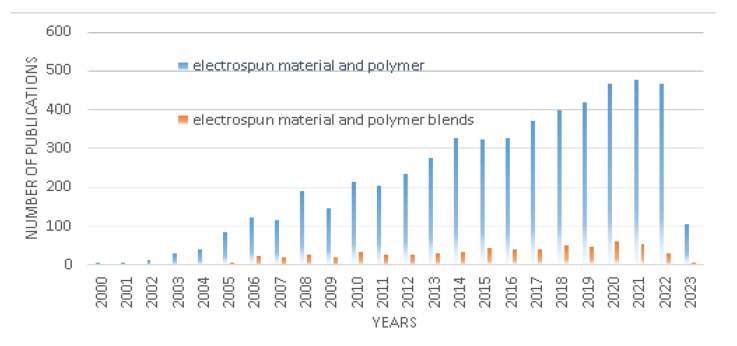
Scopus database number of publications over years comparison for the word “electrospun materials” and “polymer” (blue) to “electrospun materials” and “polymer blends” (brown).

According to Scopus database, electrospun materials have major applications in the field of materials science, chemistry, chemical engineering, physics and astronomy, biochemistry, genetics and molecular biology, and medicine. The words “electrospun material” and “polymer” have been checked in the Scopus database with title, abstract, and keywords over the years, and they have been compared to databases with a search for the words “electrospun material” and “polymer blends”. This comparison is presented in Figure 7. As can be seen, the number of reports published within the last 23 years increased in both cases. However, the search for the words “electrospun material” and “polymer blends” is much smaller than for the words “electrospun material” and “polymer”.

## 8. Conclusions and Future Outlooks

Electrospun materials have attracted great attention due to their interdisciplinary applications. In this review paper, we have focused on electrospun materials for major applications in the biomedical field, as supercapacitors, and in water treatment in the form of metal ion adsorption of heavy metals. In the case of biomedical applications, electrospun materials are used to treat cancer, coronary heart disease, and diabetic foot ulcers. Electrospun materials also have applications to control bleeding, wound healing, and in drug delivery systems. We have noticed that electrospun polymer blends have an appreciable role in biomedical applications, but their application as supercapacitors is also increasing because fossil fuel sources are depleting, and the world is focusing on energy storage devices. Electrospun materials based on polymer blends can be used to maintain the high-level performance of supercapacitors. Blends of carbon nanofibers, polysulfone, and polyacrylonitrile have key importance in supercapacitors applications. Electrospun polymer materials enhanced the adsorption capacity of heavy metals by following the metal ion adsorption technique. Blends of polyacrylonitrile and polyether sulfone have promising contributions to heavy metal ion adsorption for water treatment.

In the future, the treatment of cancer-affected cells of the human body with the electrospun polymer and biopolymer blend needs to be especially elaborated. Electrospun materials for applications in supercapacitors to achieve higher power density, maximum energy power, high capacitance, and thermal stability should be also developed. Moreover, the application of electrospun materials for effective adsorption of heavy metal ions and heavy metals is being more and more expected. 

## Data Availability

No new data were created or analyzed in this study. Data sharing is not applicable to this article.
